# Functional recovery priorities and community rehabilitation service preferences of spinal cord injury individuals and caregivers of Chinese ethnicity and cultural background

**DOI:** 10.3389/fneur.2022.941256

**Published:** 2022-08-03

**Authors:** Chor Yin Lam, Paul Aarne Koljonen, Christopher Chun Hei Yip, Ivan Yuen Wang Su, Yong Hu, Yat Wa Wong, Kenneth Man Chee Cheung

**Affiliations:** ^1^Department of Orthopaedics and Traumatology, School of Clinical Medicine, Li Ka Shing Faculty of Medicine, The University of Hong Kong, Pokfulam, Hong Kong SAR, China; ^2^Department of Orthopaedics and Traumatology, Queen Mary Hospital, Hospital Authority, Hong Kong, Hong Kong SAR, China; ^3^School of Clinical Medicine, Li Ka Shing Faculty of Medicine, The University of Hong Kong, Pokfulam, Hong Kong SAR, China; ^4^Head Office, SAHK, Hong Kong, Hong Kong SAR, China

**Keywords:** spinal cord injuries, quadriplegia, paraplegia, surveys and questionnaires, rehabilitation, community rehabilitation, rehabilitation technology, caregiver

## Abstract

**Introduction:**

Spinal cord injury (SCI) causes significant and permanent disability affecting motor, sensory and autonomic functions. We conducted a survey on the priorities of functional recovery and preferences for community rehabilitation services in a cohort of Chinese individuals with SCI as well as the primary caregivers. The study also investigated their views on advanced technology and research.

**Methods:**

An online platform with a self-administered questionnaire was used to collect the opinions of clients that received services from an SCI follow-up clinic, a self-help association, or a non-government organization from 1 September−31 December 2021.

**Results:**

Eighty-seven subjects (74 individuals with SCI−48 tetraplegic, 26 paraplegic, and 13 caregivers) responded to the survey. Recovery of arm/hand function was given the highest priority among tetraplegics, followed by upper trunk/body strength and balance, and bladder/bowel function. Sexual function had a significant lower ranking than all priority areas except normal sensation (*p* < 0.05). Paraplegics viewed bladder/bowel function as the most important area of functional recovery, followed by walking movement, upper trunk/body strength and balance, elimination of chronic pain, and regaining normal sensation. There was no statistically significant difference among the top priority areas (*p* > 0.05). In contrast to previous studies done in Western populations, the study revealed that sexual function was ranked as the lowest by all 3 groups of respondents (tetraplegics, paraplegics, and caregivers). The majority of participants thought community rehabilitation services were inadequate. Most of the respondents were interested to try advanced technology which would facilitate their daily life and rehabilitation. About half of the individuals with SCI thought advance in technology and research could bring significant improvement in their quality of life in the coming 10 years.

**Conclusion:**

This survey is the first study specifically looking into the recovery and rehabilitation priorities of a Chinese population of individuals with SCI. This is also the first study to investigate the priorities of the primary caregivers of SCI individuals. The findings are useful as a reference for planning of future research and provision of rehabilitation services for the SCI community locally and in other parts of China.

## Introduction

Spinal cord injury (SCI) is one of the most devastating physical ailments seen in clinical practice. The majority of cases results from high energy injuries such as road traffic accidents or industrial trauma. Due to an aging population, we are also seeing more geriatric patients suffering from incomplete cervical spinal cord injury after relatively minor trauma. These accidents can result in fractures or dislocations of the vertebral column, and subsequent compression and injury to the spinal cord (1).

SCI can also be caused by non-traumatic conditions such as infection, ischaemia, myelitis, and both primary and secondary malignancies affecting the spinal column. SCI affects all systems of the body below the level of neurological injury (2, 3), and can result in permanent impairments including motor paralysis, sensory loss, chronic neuropathic pain, bladder and bowel dysfunction, sexual dysfunction, persistent spasticity, progressive osteoporosis, and potentially life-threatening respiratory insufficiency, cardiovascular and autonomic dysfunction (4–8). Individuals with SCI are often hospitalized for prolonged periods, and costs in acute management, rehabilitation, and subsequent care remain extremely high (9).

According to the available published data, the prevalence of SCI is highest in the USA (906 per million) (10). However, due to the vast heterogeneity of healthcare systems, the accurate epidemiological data on the incidence of SCI in many countries, including China, is still lacking. The estimated annual incidence of SCI in some major cities in China ranged from 23.7 (Tianjian) (11) to 60.6 (Beijing) (12) per million. According to unpublished data from the Clinical Data Analysis and Reporting System of the Hospital Authority, which is responsible for the administration for all public hospitals in Hong Kong, the annual incidence of traumatic SCI in Hong Kong was ~28 cases per million during the 10 years from 2008 to 2017 (13).

From the perspective of clinicians, it is of utmost importance for us to understand the priorities for functional recovery of individuals with SCI, so that treatment and rehabilitation can be tailored to their needs. From a public health perspective, it is also imperative that resources in society are allocated fairly and efficiently in order to maximize the quality of life of individuals living with SCI in the community. And of course, knowing the recovery priorities will also help guide researchers to formulate basic science and clinical studies that can ultimately have a positive impact on the public policy of future care for these individuals.

Surveys on the priorities of functional recovery have been performed in the USA and Europe in 2000s (14–16). In the study by Anderson published in 2004, tetraplegics saw regaining arm and hand function as the most important, while sexual function was given the highest priority by paraplegics. Improving bladder and bowel function was of shared importance to both groups of SCI (14). Snoek et al. found that 77% of SCI individuals in the Netherlands and United Kingdom expected an important to very important improvement in quality of life if they could have enhancement of their hand function, and comparable expectation from improvement in bladder and bowel function (15). A more recent study has been conducted in India by Agarwal et al. (17), in which arm and hand function recovery was reported by most tetraplegics to be the first priority, and recovery of walking function was given the highest priority by most paraplegics. In this study, sexual function recovery was given a low priority (17).

Intuitively, we believe that the recovery priorities may inherently vary across different cultural backgrounds. To the best of our knowledge, we are not aware of any existing studies that have researched the priorities of functional recovery amongst individuals with SCI of Chinese ethnicity or cultural background. Relevant studies on the quality of life in individuals with SCI in Hong Kong have been conducted before, but they have not directly investigated on the priorities of functional recovery within their cohorts (18–20). Knowing the preference of individuals with SCI in our predominantly Chinese community would provide an important perspective when conducting research, tailoring rehabilitation services, and establishing public policy to best suit their needs. We also believe that the opinions of the primary caregivers deserve more attention, and to the best of our knowledge, this has not been carefully studied previously.

Particularly for countries with a government-led universal healthcare system (such as China, Canada, Australia, and most of Europe), sound community rehabilitation service planning plays an important role in maintenance of functions and prevention of complications. Previous studies on the models and delivery of SCI rehabilitation services have been carried out in other countries. Substantial differences in SCI care exist among different countries. Variations in funding sources, staffing ratio, intensity of therapy, and organization of services were found among spinal cord rehabilitation centers in North America, Europe and Southern Asia (21). SCI individuals in wealthier countries are usually provided with the necessary support for them to live in the community, including home environment adaptation and assistive devices prescription. The application for these services is coordinated and approved by a central administrative agency (22). The satisfaction with the available rehabilitation service has been shown to be high. For countries with lower incomes and financial resources, they may need to use an affordable low-cost and low-technology approach to achieve adequate care of SCI individuals (23, 24). Similar discrepancies are present in the provision of community rehabilitation after the stay in SCI rehabilitation units across different countries (25). Since there is a lack of information of service users' experience locally, we would also like to investigate their preference on current service and potential areas of service enhancement.

In the past decade, we have seen significant scientific breakthrough in the arena of assistive technology and robotics in rehabilitation (26–28). Literature on innovative rehabilitation technology such as virtual reality has shown that it was useful in reducing neuropathic pain (29), enhancing rehabilitation of upper limb function (30), balance, motor recovery, as well as helping to improve user morale and participation among individuals with SCI (31). In a recent systematic review, most rehabilitation service users and caregivers found that the use of robotics in motor rehabilitation were beneficial to the physical, psychological and social well-beings, and it was well-accepted (32). The availability of smart home technology also tended to increase the independence and quality of life among persons with impairment (33). However, the willingness to try these new technologies, and the expectation of SCI individuals and their caregivers of Chinese ethnicity has not been adequately reviewed in the literature.

Therefore, we have designed this study to explore the issues mentioned above in a Chinese population of individuals living with SCI in the local community of Hong Kong.

## Methods

### Study objective

Objective:

To study the priorities of functional recovery, and opinions on community rehabilitation service and advanced technology and research among SCI individuals and their caregivers.

Specific aims:

1. To investigate the priority of functional recovery in the SCI community,
2. To investigate the current usage and collect opinions on the available community rehabilitation service
3. To investigate the expectation of the effects of advanced technology and research on their quality of life in the coming 10 years

### Study population

Potential respondents in this survey included individuals who suffered from SCI and their primary caregivers. The SCI could be traumatic or non-traumatic. They were recruited through a major non-government organization which provided services to the subjects (SAHK, formerly known as Spastic Association of Hong Kong, Hong Kong SAR, China), a self-help organization (Direction Association for the Handicapped, Hong Kong SAR, China), and the Specialist Out-patient Clinic for SCI in a rehabilitation hospital (MacLehose Medical Rehabilitation Centre, Hong Kong SAR, Hong Kong) during the period 1 September−31 December 2021. Posters and pamphlets with QR-codes for access to the self-administered questionnaire on an online survey platform (Qualtrics XM, www.qualtrics.com) were distributed to the premises and clients attending services of the recruitment partners mentioned. The clients could access the online survey with computers or mobile devices. All eligible subjects gave consent to participate in the survey by checking the online agreement at the start of the survey.

Inclusion criteria:

1. Individuals with traumatic or non-traumatic SCI, or their primary caregivers
2. Age 18 or older
3. Understand written English or Chinese.

Exclusion criteria:

1. There were no other exclusion criteria for this survey.

### Study design

A self-administered online questionnaire was designed. The questionnaire was bilingual (English and traditional Chinese). Background information of the subjects, including their roles in the SCI community (individuals with SCI vs. primary caregivers), and details of the SCI, was collected. The primary means of mobility, and the time spent on daily care by their caregivers, self-exercise, and activities outside their residence were recorded. The areas of functional recovery priorities were classified into 7 categories with reference to a previous study performed by Anderson (14), namely arm/hand function, upper body/trunk strength and balance, bladder/bowel function, sexual function, elimination of chronic pain, normal sensation, and walking movement. A survey has shown that these areas are among the most commonly evaluated parameters by clinicians and researchers interested in SCI rehabilitation (34). These areas are also shown to have substantial impacts on the quality of life for SCI individuals in the literatures. Participants were then asked to arrange the priorities of these 7 areas of functional recovery in order of importance to them. Their usage and opinions on the current community rehabilitation service were also surveyed. Finally, they were asked about their willingness to try advanced technology in rehabilitation, and their expectations of the effects of advanced technology and research on their quality of life in the coming 10 years.

Approval for this study has been sought from Institutional Review Board of the University of Hong Kong/Hospital Authority Hong Kong West Cluster (reference number UW 21-480).

### Study questionnaire

The questions of the online questionnaire are available in Appendix 1. The participants needed to confirm their eligibility and gave their consents to the survey before they could proceed.

### Statistical analysis

Data obtained in the survey were analyzed with IBM® SPSS® Statistics version 26. Descriptive statistics were obtained. The median rankings of the priorities of 7 areas of functional recovery were calculated. The rankings of functional recovery priorities were tested for statistical significance with Independent-Samples Kruskal-Wallis Test. The level of significance was set at 0.05.

## Results

### Study population characteristics

A total of 87 subjects responded to the survey. Among them, 74 were individuals with SCI and 13 were primary caregivers. All subjects answered the questionnaires in traditional Chinese.

### Individuals with SCI

Among the 74 respondents, 56 (75.7%) were male and 18 (24.3%) were female. The mean age was 51.2 years (18–78). 48 (64.9%) were tetraplegic and 26 (35.1%) were paraplegic. The regions of neurological injuries were recorded. 3 (4.1%) of the respondents were not sure about which region had been injured. 33 (44.6%) individuals reported their injuries to be complete and an equal number reported their injuries to be incomplete. 8 (10.8%) of the respondents were not sure about the completeness of their injuries. The duration of time post-injury ranged from 0 to 38 years (mean 10.1 years). On average, they required 10.1 (0–24) h of care by others every day. They spent an average of 8.5 (0–28) h per week doing exercise by themselves or assisted by their caregivers. They went outside their residence for leisure activities (excluding attention of medical or rehabilitation services) for 11.1 (0–60) h per week.

The majority used wheelchairs as their primary means of mobility. 3 (4.1%) of them reported that they were bedbound and only 7 (9.3%) of the respondents could walk independently. Concerning the types of residence, 23 (31.1%) were living in private housing while 33(44.6%) were living in public housing. 11 (14.9%) were living in transitional facilities waiting for arrangement of their permanent residence.

#### Primary caregivers

Among the primary caregivers for SCI individuals, 5 (38.5%) were male and 8 (61.5%) were female. Their mean age was 50.8 years (36–66). The majority of them are taking care of individuals with the neurological level of injury at the cervical spine (7, 54.8%). The mean duration of taking care of the individual was 8 (3–34) years, and they spent 9.9 (1–24) h taking care of the individual per day. The majority of the individuals under care used power wheelchairs for mobility (6, 46.2%). 3 (23.1%) individuals used manual wheelchairs and another 3 (23.1%) used wheelchairs controlled or propelled by the caregivers. Only 1 (7.7%) of the individuals under care was able to walk independently. These individuals spent an average of 9.5 (1–28) h per week doing exercise by themselves or assisted by their caregivers. They went outside their residence for leisure activities (excluding attention of medical or rehabilitation services) for 13 (0–21) h per week.

The details of the survey participants are shown in Table 1.

**Table 1:** Characteristics of survey participants.

**Individuals with SCI**	**Paraplegic**	**Tetraplegic**	**All**
**Number**	26 (35.1%)	48 (64.9%)	74 (100%)
**Sex**			
Male	18 (69.2%)	38 (79.2%)	56 (75.7%)
Female	8 (30.8%)	10 (20.8%)	18 (24.3%)
**Average age**	52.2 (18–69)	50.1 (24–78)	51.9 (18–78)
**Average years post-injury**	11.4 (0–38)	9.37 (0–29)	10.1 (0–38)
**Region injured**			
Cervical	0	48 (100%)	48 (64.9%)
Thoracic	13 (50%)	0 (0%)	13 (17.6%)
Lumbosacral	10 (38.5%)	0 (0%)	10 (13.5%)
Unknown	3 (11.5%)	0 (0%)	3 (4.0%)
**Severity**			
Complete injury	10 (38.5%)	23 (47.9%)	33 (44.6%)
Incomplete injury	12 (46.2%)	21 (43.8%)	33 (44.6%)
Unknown	4 (15.3%)	4 (8.3%)	8 (10.8%)
**Primary mobility**			
Bedbound	0 (0%)	3 (6.3%)	3 (4.1%)
Power wheelchair	12 (46.2%)	28 (58.3%)	40 (54.1%)
Manual wheelchair	7 (26.9%)	8 (16.7%)	15 (20.3%)
Wheelchair controlled/propelled by caregiver	5 (19.2%)	4 (8.3%)	9 (12.2%)
Walk with aid independently	2 (7.7%)	4 (8.3%)	6 (8.0%)
Walk without aids independently	0 (0%)	1 (2.1%)	1 (1.3%)
**Current residence**			
Private Housing	8 (30.8%)	15 (31.3%)	23 (31.1%)
Public Housing	11 (42.3%)	22 (45.8%)	33 (44.6%)
Private institution	0 (0%)	1 (2.1%)	1 (1.4%)
Government/subvented institution	2 (7.7%)	1 (2.1%)	3 (4.0%)
Transitional housing	4 (15.4%)	7 (14.6%)	11 (14.9%)
Others	1 (3.8%)	2 (4.2%)	3 (4.0%)
**Average no. of hours**			
Cared by caregiver per day	7.9 (0–24)	11.3 (0–24)	10.1 (0–24)
Exercise per week	7.5 (0–24)	9.0 (0–28)	8.5 (0–28)
Going out per week	9.8 (0–56)	11.8 (0–60)	11.1 (0–60)
**Caregivers**			
**Number**	13		
**Sex**			
Male	5 (38.5%)		
Female	8 (61.5%)		
**Age**	50.8 (36–66)		
**Average duration taking care of SCI individual (year)**	11.7 (3–34)		
**Individuals with SCI under care**			
**Region injured**			
Cervical	7 (53.8%)		
Thoracic	2 (15.4%)		
Lumbosacral	4 (30.8%)		
Unknown	0 (0%)		
**Severity**			
Complete injury	5 (38.5%)		
Incomplete injury	3 (23.1%)		
Unknown	5 (38.5%)		
**Primary mobility**			
Bedbound	0 (0%)		
Power wheelchair	6 (46.2%)		
Manual wheelchair	3 (23.1%)		
Wheelchair controlled/propelled by caregiver	3 (23.1%)		
Walk with aid independently	0 (0%)		
Walk without aids independently	1 (7.7%)		
**Average no. of hours spent**			
Cared by caregiver per day	9.9 (1–24)		
Exercise per week	9.5 (1–28)		
Going out per week	13.0 (0–21)		

### Priorities of functional recovery

#### Individuals with SCI

For tetraplegic individuals, recovery of arm/hand function was given the highest priority (median ranking 2), followed by upper trunk/body strength and balance, and bladder/bowel function (both having median ranking 3). Walking movement had a median ranking of 4. Elimination of chronic pain and normal sensation were both given a median ranking of 5. Sexual function was given the lowest median ranking of 7. Independent-Samples Kruskal-Wallis Test showed no statistically significant difference among the top 3 priority areas (*p* > 0.05). The ranking for recovery of arm/hand function was statistically significantly higher than sexual function, elimination of chronic pain, normal sensation, and walking movement (*p* < 0.05). Upper trunk/body strength and balance was rated to be significantly higher than sexual function, elimination of chronic pain, and normal sensation (*p* < 0.05). Bladder/bowel function was rated to be significantly higher than sexual function, elimination of chronic pain, and normal sensation (*p* < 0.05). Recovery of sexual function had a significantly lower ranking than all other priority areas except normal sensation (*p* < 0.05).

Paraplegic individuals viewed bladder/bowel function as the most important area of functional recovery (median ranking 2), followed by walking movement (median ranking 3), upper trunk/body strength and balance, elimination of chronic pain, and normal sensation (all having median ranking 4). Arm/hand function, and sexual function were rated the lowest with both having median rankings of 5. Independent-Samples Kruskal-Wallis Test showed no statistically significant difference among bladder/bowel function, walking movement, upper trunk/body strength and balance, and elimination of chronic pain (*p* > 0.05). The ranking for bladder/bowel function was rated to be significantly higher than sexual function, normal sensation, and arm/hand function (*p* < 0.05). Sexual function was also ranked significantly lower than walking movement (*p* < 0.05).

When the priority areas of functional recovery between the 2 groups were compared, arm/hand function was rated significantly higher by tetraplegic (median ranking 2) than paraplegic (median ranking 5) individuals (*p* < 0.05).

#### Primary caregivers

The primary caregivers of individuals with SCI in fact had their opinions similar to the tetraplegic individuals. The recovery of arm/hand function, and upper trunk/body strength and balance were given the highest priority (both having median ranking 2), followed by bladder/bowel function (median ranking 3). Walking movement was given a median ranking of 4, while both elimination of chronic pain and normal sensation had median rankings of 5. Again, sexual function was given the lowest median ranking of 7. There was no statistically significant difference among the top 4 priority areas when tested with Independent-Samples Kruskal-Wallis Test (*p* > 0.05). The ranking of recovery of arm/hand function was rated to be significantly higher than sexual function, elimination of chronic pain, and normal sensation (*p* < 0.05). Sexual function was also ranked significantly lower than upper trunk/body strength and balance, and bladder/bowel function (*p* < 0.05).

**Figure 1:**
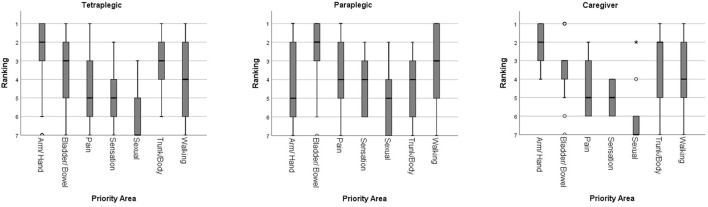
Overall rankings of the priority areas of functional recovery.

The overall rankings of the priority areas of functional recovery in each group are shown in Figure 1. The summary of statistical tests is shown in Tables 2, 3.

**Table 2:** Summary of statistical findings in tetraplegic, paraplegic, and caregivers.

**Individuals with SCI**	**High priority areas**	**Versus**	***p*-value**
Tetraplegic	Arm/hand function	Walking	0.016
		Chronic pain	0.000
		Normal sensation	0.000
		Sexual function	0.000
	Trunk/body strength	Chronic pain	0.040
		Normal sensation	0.003
		Sexual function	0.000
	Bladder/bowel function	Normal sensation	0.008
		Sexual function	0.000
	Walking	Sexual function	0.000
	Chronic pain	Sexual function	0.004
	**High priority areas**	>**Versus**	>***P*****-value**
Paraplegic	Bladder/bowel function	Normal sensation	0.049
		Arm/hand function	0.005
		Sexual function	0.000
	Walking	Sexual function	0.007
**Caregiver**	**High priority areas**	**Versus**	***P*** **-value**
	Arm/hand function	Chronic pain	**0.027**
		Normal sensation	0.003
		Sexual function	0.000
	Trunk/body strength	Sexual function	0.019
	Bladder/bowel function	Sexual function	0.027

*Independent-Samples Kruskal-Wallis Test (only statistically significant findings shown)*.

**Table 3:** Summary of statistical findings in priorities of functional recovery between tetraplegic and paraplegic.

**Priority areas**	**Median ranking**	***p*-value**
Arm/hand function	Tetraplegic: 2	0.000
	Paraplegic: 5	

*Independent-Samples Kruskal-Wallis Test (only statistically significant findings shown)*.

#### Subgroups analysis according to gender, time post-injury, and age

The overall rankings of the priority areas of functional recovery among the tetraplegic and paraplegic individuals were further analyzed according to gender, time post-injury and age. The summary of the statistical tests of these subgroups is shown in Table 4. Comparison between the 2 different subgroups within each category was performed with Independent-Samples Kruskal-Wallis Test and the summary is shown in Table 5.

**Table 4:** Summary of statistical findings in subgroups of SCI individuals.

**Individuals with SCI**	**High priority areas**	**Versus**	***p*-value**
Tetraplegic Male (*n* = 38)	Arm/hand function	Chronic pain	0.008
		Normal sensation	0.000
		Sexual function	0.000
	Bladder/bowel function	Normal sensation	0.008
		Sexual function	0.000
	Trunk/body strength	Normal sensation	0.023
		Sexual function	0.000
	Walking	Sexual function	0.001
Tetraplegic Female (*n* = 10)	Arm/hand function	Chronic pain	0.012
		Normal sensation	0.001
		Sexual function	0.000
	Trunk/body strength	Sexual function	0.000
	Bladder/bowel function	Sexual function	0.018
	Walking	Sexual function	0.027
Paraplegic Male (*n* = 18)	Bladder/bowel function	Arm/hand function	0.008
		Sexual function	0.001
	Walking	Sexual function	0.034
Paraplegic Female (*n* = 8)	No statistically significant difference found among priority areas (*p >* 0.05)		
Tetraplegic 3 years or less post-injury (*n* = 16)	Walking	Sexual function	0.000
	Arm/hand function	Sexual function	0.003
	Bladder/bowel function	Sexual function	0.001
	Trunk/body strength	Sexual function	0.002
Tetraplegic more than 3 years post-injury (*n* = 32)	Arm/hand function	Walking	0.000
		Chronic pain	0.000
		Normal sensation	0.000
		Sexual function	0.000
	Trunk/body strength	Chronic pain	0.047
		Normal sensation	0.004
		Sexual function	0.000
	Bladder/bowel function	Normal sensation	0.013
		Sexual function	0.000
Paraplegic 3 years or less post-injury (*n* = 9)	Bladder/bowel function	Sexual function	0.001
	Walking	Sexual function	0.002
Paraplegic more than 3 years post-injury (*n* = 17)	No statistically significant difference found among priority areas (*p >* 0.05)		
Tetraplegic 40 or younger (*n* = 10)	Arm/hand function	Chronic pain	0.040
		Normal sensation	0.040
		Sexual function	0.002
Tetraplegic older than 40 (*n* = 38)	Arm/hand function	Chronic pain	0.001
		Normal sensation	0.000
		Sexual function	0.000
	Trunk/body strength	Normal sensation	0.023
		Sexual function	0.000
	Bladder/bowel function	Normal sensation	0.034
		Sexual function	0.000
	Walking	Sexual function	0.000
	Chronic pain	Sexual function	0.008
Paraplegic 40 or younger (*n* = 6)	No statistically significant difference found among priority areas (*p >* 0.05)		
Paraplegic older than 40 (*n* = 20)	Bladder/bowel function	Chronic pain	0.020
		Normal sensation	0.002
		Arm/hand function	0.000
		Sexual function	0.000
	Walking	Sexual function	0.000

*Independent-Samples Kruskal-Wallis Test (only statistically significant findings shown)*.

**Table 5:** Summary of statistical findings in tetraplegic and paraplegic according to gender, time post-injury, and age.

**Subgroups**	**Priority areas**	**Median ranking**	***p*-value**
**Tetraplegic**			
Gender	No statistically significant difference (*p >* 0.05)		
Time Post-injury	Arm/hand function	3 years or less: 3 More than 3 years: 2	0.025
	Walking	3 years or less: 2.5 More than 3 years: 4	0.024
Age	No statistically significant difference (*p >* 0.05)	
**Paraplegic**			
Gender	No statistically significant difference (*p >* 0.05)		
Time Post-injury	Sexual function	3 years or less: 7 More than 3 years: 5	0.033
Age	Bladder/bowel function	40 or younger: 5.5 Older than 40: 1.5	0.022
	Sexual function	40 or younger: 3.5 Older than 40: 6	0.004

*Independent-Samples Kruskal-Wallis Test (only statistically significant findings shown)*.

##### Subgroup analysis according to gender

There were 38 male and 10 female tetraplegic individuals. Both genders gave the highest priority (median ranking male:2, female:1) to arm/hand function, followed by upper trunk/body strength and balance (median ranking both genders:3). Bladder/bowel function was rated higher by male (median ranking 3 vs. 4.5 in female). Walking movement was given a higher ranking by female (median ranking 3.5 vs. 4 in male). Normal sensation (median ranking male:5, female:4.5) and elimination of chronic pain (median ranking male:5, female:6) and were put lower in priority. Both genders gave sexual function the lowest median ranking of 7. Independent-Samples Kruskal-Wallis Test showed no statistically significant difference among the top 4 priority areas (*p* > 0.05) in both genders. Recovery of arm/hand function was given significantly higher ranking than sexual function, elimination of chronic pain, and normal sensation (*p* < 0.05). Sexual function had a significantly lower ranking than all other priority areas except elimination of chronic pain and normal sensation (*p* < 0.05).

There were 18 male and 8 female paraplegic individuals. Both genders gave the highest priority (median ranking both sexes:2) to bladder/bowel function, followed by walking movement (median ranking male: 2.5, female: 3), upper trunk/body strength and balance, elimination of chronic pain, and normal sensation (median ranking male: 3.5–5, female: 4–4.5). Arm/hand function (median ranking male: 5.5, female: 5), and sexual function (median ranking male: 5, female: 6) were rated the lowest. Testing with Independent-Samples Kruskal-Wallis Test showed no statistically significant difference among bladder/bowel function, walking movement, upper trunk/body strength and balance, normal sensation, and elimination of chronic pain (*p* > 0.05). The ranking for bladder/bowel function was rated significantly higher than sexual function and arm/hand function (*p* < 0.05). Sexual function was also ranked significantly lower than walking movement (*p* < 0.05).

**Figure 2:**
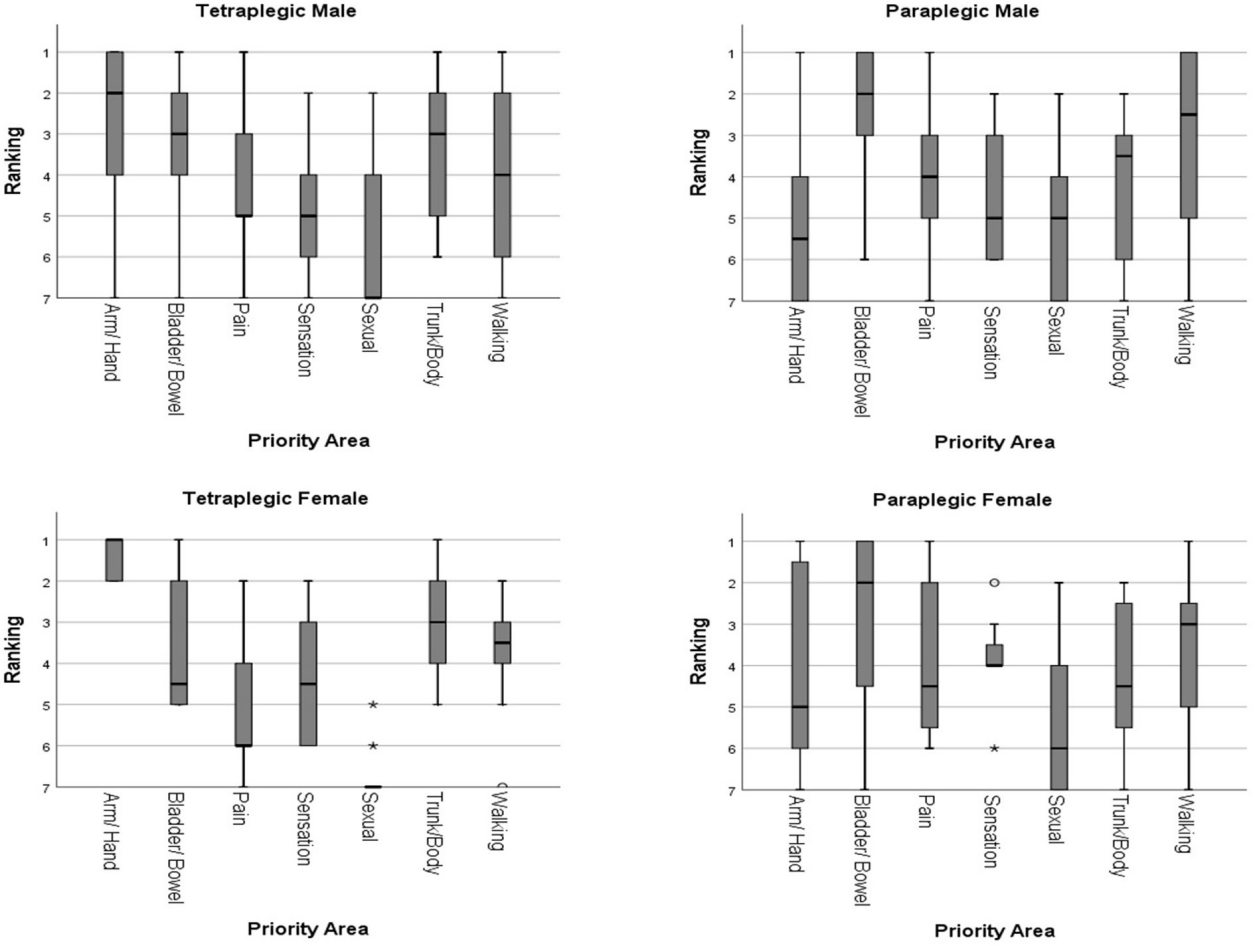
Rankings of the priority areas of functional recovery according to gender.

Overall, the priority rankings were similar for both genders. No statistically significant difference was found between the different genders (*p* > 0.05). The rankings of the priority areas of functional recovery in different genders are shown in Figure 2.

##### Subgroup analysis according to time post-injury

The SCI individuals were divided into 2 groups according to time post-injury (3 years or less, and more than 3 years). We have arbitrarily taken 3 years post-injury as a dividing timepoint, as this would have allowed all SCI individuals ample time for full physical and psychological adaptation to their disabilities after neurological plateau, as well as to be well-settled in their respective communities. Among the tetraplegic individuals, 16 were 3 years or less post-injury and 32 were more than 3 years post-injury. For tetraplegic individuals of 3 years or less post-injury, walking movement (median ranking 2.5) and arm/hand function (median ranking 3) were rated the highest priorities, followed by bladder/bowel function, upper trunk/body strength and balance, elimination of chronic pain, and normal sensation (median ranking 3.5–5). Sexual function had the lowest median ranking of 7. Independent-Samples Kruskal-Wallis Test showed no statistically significant difference among the top 6 priority areas (*p* > 0.05). Recovery of sexual function had a significant lower ranking than all other priority areas except elimination of chronic pain and normal sensation (*p* < 0.05).

For tetraplegic individuals of more than 3 years post-injury, the result was similar to the overall priority ranking of tetraplegic individuals. Recovery of arm/hand function was given the highest priority (median ranking 2), followed by upper trunk/body strength and balance, and bladder/bowel function (both having median ranking 3). Walking movement had a median ranking of 4. Elimination of chronic pain and normal sensation were both given a median ranking of 5. Sexual function was given the lowest median ranking of 7. Independent-Samples Kruskal-Wallis Test showed no statistically significant difference among the top 3 priority areas (*p* > 0.05). The ranking for recovery of arm/hand function was significantly higher than sexual function, elimination of chronic pain, normal sensation, and walking movement (*p* < 0.05). Upper trunk/body strength and balance was rated to be significantly higher than sexual function, elimination of chronic pain, and normal sensation (*p* < 0.05). Bladder/bowel function was rated to be significantly higher than sexual function, and normal sensation (*p* < 0.05). Recovery of sexual function had a significant lower ranking than all other priority areas except elimination of chronic pain, walking movement and normal sensation (*p* < 0.05). Compared with the group of 3 years or less post-injury, tetraplegic individuals more than 3 years post-injury gave arm/hand function significantly higher priority, and walking movement significantly lower priority (both *p* < 0.05).

Among the paraplegic individuals, 9 were 3 years or less post-injury, and 17 were more than 3 years post-injury. For paraplegic individuals of 3 years or less post-injury, bladder/bowel function and walking movement were given the highest ranking (median ranking 2), followed by upper trunk/body strength and balance, elimination of chronic pain and normal sensation (all 3 having median ranking 4). Arm/hand function was given a median ranking of 5. Sexual function had the lowest median ranking of 7. Independent-Samples Kruskal-Wallis Test showed that there was no significant difference between bladder/bowel function and walking movement (*p* > 0.05). The top 2 priorities had statistically significant higher ranking than sexual function (*p* < 0.05).

**Figure 3:**
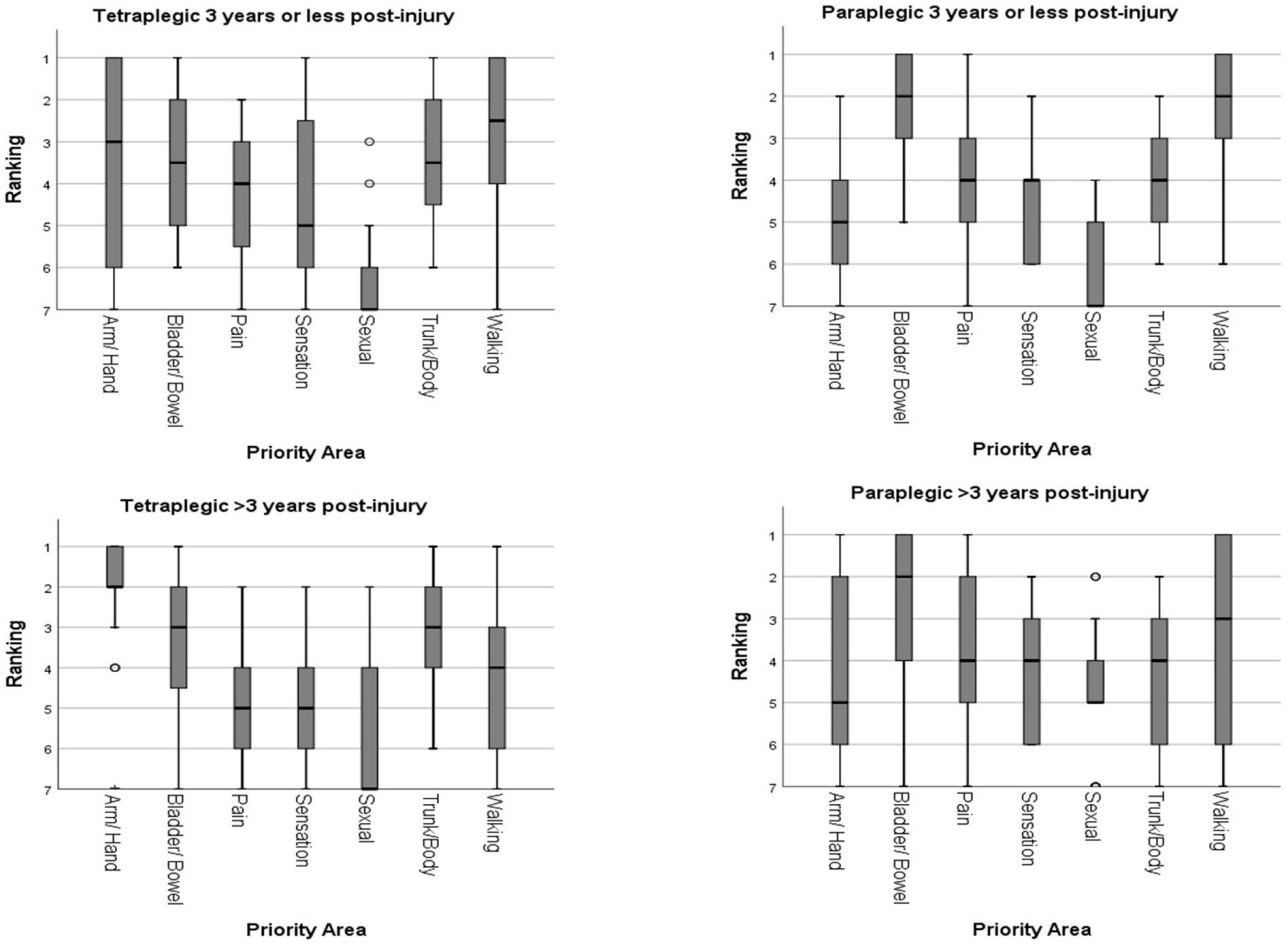
Rankings of the priority areas of functional recovery according to different time post-injury.

For paraplegic individuals of more than 3 years post-injury, the highest median rankings were given to bladder/bowel function and walking movement (median ranking 2 and 3, respectively). Upper trunk/body strength and balance, elimination of chronic pain and normal sensation all had a median ranking of 4. Arm/hand function and sexual function were both given median rankings of 5. No statistically significant differences were found among all 7 priority areas (*p* > 0.05). Compared with the group 3 years or less post-injury, paraplegics individuals more than 3 years post-injury gave sexual function significantly higher priority (both *p* < 0.05). The rankings of the priority areas of functional recovery according to different time post-injury are shown in Figure 3.

##### Subgroup analysis according to age

The SCI individuals were divided into 2 groups according to age (40 years old or younger, and older than 40).

Among the tetraplegic individuals, 10 were 40 years old or younger, and 38 were older than 40. For tetraplegic individuals of age 40 years or younger, arm/hand function was given the highest priority (median ranking 1), followed by upper trunk/body strength and balance (median ranking 2.5), and bladder/bowel function (median ranking 3). Walking movement had a median ranking of 4. Elimination of chronic pain and normal sensation were both given a median ranking of 5. Sexual function was given the lowest median ranking of 6. Independent-Samples Kruskal-Wallis Test showed that recovery of arm/hand function had statistically significant higher priority than elimination of chronic pain, normal sensation, and sexual function (*p* < 0.05). There were no other statistically significant differences among other priority areas.

For tetraplegic individuals older than 40, arm/hand function was again given the highest priority (median ranking 2), followed by upper trunk/body strength and balance, and walking movement (both median ranking 3). Bladder/bowel function had a slightly lower median ranking of 3.5. Elimination of chronic pain and normal sensation were both given a median ranking of 5. Sexual function was given the lowest median ranking of 7. Independent-Samples Kruskal-Wallis Test showed no statistically significant difference among the top 4 priority areas (*p* > 0.05). The ranking for recovery of arm/hand function was statistically significantly higher than sexual function, elimination of chronic pain, and normal sensation (*p* < 0.05). Upper trunk/body strength and balance, and bladder/bowel function were rated to be significantly higher than sexual function, and normal sensation (*p* < 0.05). Recovery of sexual function had a significant lower ranking than all other priority areas except normal sensation (*p* < 0.05). Overall, the priority rankings were similar for both age groups of tetraplegic individuals. No statistically significant difference was found (*p* > 0.05).

Among the paraplegic individuals, 6 were 40 years old or younger, and 20 were older than 40. For paraplegic individuals of age 40 years or younger, elimination of chronic pain, normal sensation, and sexual function shared the top 3 priorities with median rankings of 3.5. Walking movement had a median ranking of 4. It was followed by arm/hand function, and upper trunk/body strength and balance (both median ranking 4.5). Bladder/bowel function was rated the lowest with a median ranking of 6. No statistically significant differences were found (*p* > 0.05) among all 7 priority areas.

For paraplegic individuals older than 40, bladder/bowel function had the highest median ranking of 1.5. It was followed by walking movement (median ranking 3), upper trunk/body strength and balance (median ranking 3.5), elimination of chronic pain (median ranking 4), normal sensation (median ranking 4.5). Arm/hand function and sexual function were rated to have the lowest priority (median ranking 5.5 and 6, respectively). Independent-Samples Kruskal-Wallis Test showed that there was no statistically significant difference among the top 3 priority areas (*p* > 0.05). Bladder/bowel function had significantly higher priority than arm/hand function, elimination of chronic pain, normal sensation, and sexual function (*p* < 0.05). Walking movement was rated significantly higher than sexual function (*p* < 0.05). There were no other statistically significant differences among other priority areas.

**Figure 4:**
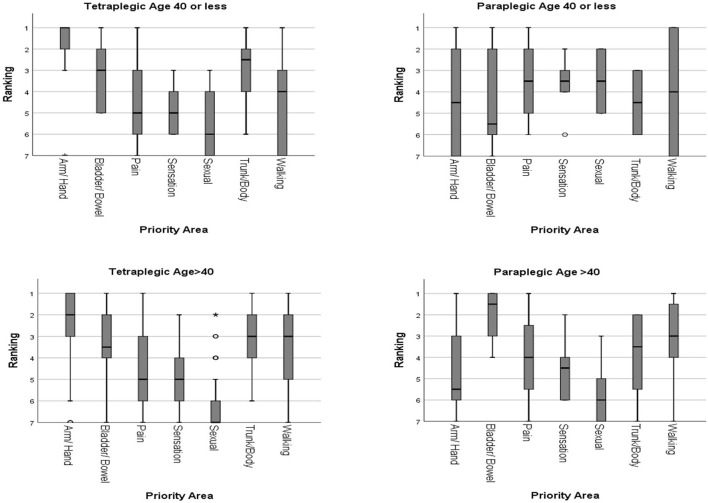
Rankings of the priority areas of functional recovery according to age.

The younger age group of paraplegic individuals rated significantly higher priority for sexual function than the older age group (*p* < 0.05). The rankings of the priority areas of functional recovery according to age are shown in Figure 4.

### Use of community rehabilitation services

#### Individuals with SCI

56 (64.4%) of respondents were actively using community rehabilitation services. Community rehabilitation services, either day or home services, provided by non-governmental organizations were the most commonly used (47, 83.9% of users). Among different disciplines of services, physiotherapy (50, 89.3% of users) and occupational therapy (43, 76.7% of users) were the most popular, followed by nursing care (24, 42.8% of users) and general care service (18, 32.1% users). Only 13 (14.9%) respondents thought the current community rehabilitation services were adequate while 50 (57.5%) commented that they were inadequate. Most individuals would like to see increase in physiotherapy (44, 50.6%), occupational therapy (28, 32.2%), and nursing care (30, 34.5%) services. Traditional Chinese medicine treatment and robotic rehabilitation were mentioned as additional services they would like to receive.

#### Primary caregivers

10 of the 13 caregivers (76.9%) in the survey were supported by community rehabilitation services. Again, service provided by non-governmental organizations were the most commonly used (7, 70% of users). Like the utilization patterns of the individuals with SCI, physiotherapy (6, 60% of users) and occupational therapy (6, 60% of users) were the most popular, followed by nursing care (4, 40% of users) and general care service (4, 40% users). 11 out of 13 (84.6%) caregivers thought that the current community rehabilitation service was inadequate. Physiotherapy (8, 61.5%), occupational therapy (7, 53.8%) and general care service (5, 38.5%) were the top 3 areas which most of them would like to see increase.

The details of opinions from the SCI individuals and caregivers on community rehabilitation services are shown in Table 6.

**Table 6:** Opinions on community rehabilitation services.

**Individuals with SCI**	**Paraplegic**	**Tetraplegic**	**All**
**Do you think the service is important?**			
Very important	9 (34.6%)	26 (56.5%)	35 (47.3%)
Fairly important	3 (11.5%)	11 (22.9%)	14 (18.9%)
Important	8 (30.9%)	8 (16.7%)	16 (21.6%)
Slightly important	3 (11.5%)	1 (2.1%)	4 (5.4%)
Not important	0 (0%)	0 (0%)	0 (0%)
No opinion	3 (11.5%)	2 (4.2%)	5 (6.8%)
**Adequacy of services**			
Yes	4 (15.4%)	9 (18.7%)	13 (17.6%)
No	17 (65.4%)	33 (68.8%)	50 (67.6%)
No opinion	5 (19.2%)	6 (12.5%)	11 (14.8%)
**If no, service needed to be strengthened**			
Physiotherapy	14 (82.4%)	30 (90.9%)	44 (88.0%)
Occupational therapy	6 (35.3%)	22 (66.7%)	28 (56.0%)
Speech therapy	3 (17.6%)	3 (9.1%)	6 (12.0%)
Nursing	9 (52.9%)	21 (63.6%)	30 (60.0%)
General care	6 (35.3%)	17 (51.5%)	23 (46.0%)
Others	2 (11.8%)	0 (0%)	2 (4.0%)
**Using community rehabilitation services**			
Yes	17 (65.4%)	39 (81.3%)	56 (75.7%)
No	9 (34.6%)	9 (18.7%)	18 (24.3%)
**Current users**			
**Service providers**			
Public outreach service	1 (5.9%)	2(5.1%)	3 (5.4%)
Public outpatient/day service	5 (29.4%)	7 (17.9%)	12 (21.4%)
Non-governmental organization outreach	5 (29.4%)	11 (28.2%)	16 (28.6%)
Non-governmental organization outpatient/day	10 (58.8%)	20 (51.3%)	30 (53.6%)
Private service	3 (17.6%)	4 (10.3%)	7 (12.5%)
**Service used**			
Physiotherapy	14 (82.4%)	36 (92.3%)	50 (89.3%)
Occupational therapy	11 (64.7%)	32 (82.1%)	43 (76.8%)
Speech therapy	1 (5.9%)	1 (5.9%)	2 (3.6%)
Nursing	3 (17.6%)	21 (53.8%)	24 (42.9%)
General care	5 (29.4%)	13 (33.3%)	18 (32.1%)
Others	1 (5.9%)	0 (0%)	1 (1.8%)
**Non-users**			
**Would you like to use the service if available?**			
Yes	4 (44.4%)	6 (66.7%)	10 (55.6%)
Maybe	5 (55.6%)	3 (33.3%)	8 (44.4%)
No	0 (0%)	0 (0%)	0 (0%)
**If yes/maybe, what service?**			
Physiotherapy	9 (100%)	8 (88.9%)	17 (94.4%)
Occupational therapy	4 (44.4%)	4 (44.4%)	8 (44.4%)
Speech therapy	0 (0%)	0 (0%)	0 (0%)
Nursing	5 (55.6%)	3 (33.3%)	8 (44.4%)
General care	4 (44.4%)	3 (33.3%)	7 (38.9%)
Others	1 (11.1%)	0 (0%)	1 (5.6%)
**Caregivers**			
**Do you think the service is important?**			
Very important	10 (76.9%)		
Fairly important	1 (7.7%)		
Important	2 (15.4%)		
Slightly important	0 (0%)		
Not important	0 (0%)		
No opinion	0 (0%)		
**Adequacy of services**			
Yes	0 (0%)		
No	11 (84.6%)		
No opinion	2 (15.4%)		
**If no, service needed to be strengthened**			
Physiotherapy	8 (72.7%)		
Occupational therapy	7 (63.6%)		
Speech therapy	0 (0%)		
Nursing	4 (36.4%)		
General care	5 (45.5%)		
Others	0 (0%)		
**Supported by community rehabilitation services**			
Yes	10 (76.9%)		
No	3 (23.1%)		
**Current users**			
**Service providers**			
Public outreach service	2 (20.0%)		
Public outpatient/day service	2 (20.0%)		
Non-governmental organization outreach	6 (60.0%)		
Non-governmental organization outpatient/day service	4 (40.0%)		
Private service	0 (0%)		
**Service used**			
Physiotherapy	6 (60.0%)		
Occupational therapy	6 (60.0%)		
Speech therapy	0 (0%)		
Nursing	4 (40.0%)		
General care	4 (40.0%)		
Others	0 (0%)		
**Non-users**			
**Would you like to use the service if available?**			
Yes	2 (66.7%)		
Maybe	1 (33.3%)		
No	0 (0%)		
**If yes/maybe, what service?**			
Physiotherapy	3 (100%)		
Occupational therapy	2 (66.7%)		
Speech therapy	0 (0%)		
Nursing	3 (100%)		
General care	3 (100%)		
Others	0 (0%)		

### Views on the advance in technology and research

#### Individuals with SCI

Concerning the topic of advancement in technology and research, 73 individuals with SCI responded to this part of the survey. Most of them (66, 75.9% of respondents) would like to try advanced technology in rehabilitation, in particular robotics and smart home modifications.

A significant proportion (47.9% of tetraplegics and 50.0% of paraplegics) of SCI individuals responded with optimism when asked if advanced technology could directly improve their quality of life within the coming 10 years. An average of 20.6% of the respondents thought that a significant impact was unlikely, while 31.1% were unsure. When compared with paraplegics, tetraplegic individuals responded with a relatively more guarded outlook−29.2% of all tetraplegics, compared with 3.8% in paraplegics—thought that a substantial breakthrough was unlikely.

#### Caregivers

All 13 caregivers responded that they would like the individuals they took care of to try advanced technology in rehabilitation. They are also more optimistic than the individuals with SCI that advance in technology and research could bring significant improvement in their quality of life in the coming 10 years. 69.3% of the caregivers thought that the goal could likely be achieved.

The details of the views on the advance in technology are shown in Table 7.

**Table 7:** Views on the advancement in technology and research.

**Individuals with SCI**	**Paraplegic**	**Tetraplegic**	**All**
**Would you try advanced technology if available in the community?**			
Yes	23 (88.5%)	43 (89.6%)	66 (89.2%)
Maybe	3 (11.5%)	3 (6.3%)	6 (8.1%)
No	0 (0%)	2 (4.1%)	2 (2.7%)
**The chance that advances in technology and research in the coming 10 years can significantly improve the QOL of SCI individuals**			
Extremely likely (>80%)	4 (15.4%)	12 (25.0%)	16 (21.6%)
Somewhat likely (60–80%)	9 (34.6%)	11 (22.9%)	20 (27.0%)
Neither likely nor unlikely (40–60%)	12 (46.2%)	11 (22.9%)	23 (31.1%)
Somewhat unlikely (20–40%)	1 (3.8%)	10 (20.8%)	11 (14.9%)
Extremely unlikely (<20%)	0 (0%)	4 (8.4%)	4 (5.4%)
**Caregivers**			
**Would you try advanced technology if available in the community?**			
Yes	13 (100%)		
Maybe	0 (0%)		
No	0 (0%)		
**The chance that advances in technology and research in the coming 10 years can significantly improve the QOL of SCI individuals**			
Extremely likely (>80%)	4 (30.8%)		
Somewhat likely (60–80%)	5 (38.5%)		
Neither likely nor unlikely (40–60%)	3 (23.1%)		
Somewhat unlikely (20–40%)	1 (7.6%)		
Extremely unlikely (<20%)	0 (0%)		

## Discussion

This survey utilized an online platform to study the opinions of the SCI community in Hong Kong. All participants of this survey were of Chinese ethnicity and cultural background. It demonstrated the varying priorities of functional recovery areas between tetraplegic and paraplegic individuals.

Participants could use either mobile devices or computers to complete the survey. They could respond to the survey at times and places which they found to be most convenient, and there was no potential embarrassment on answering questions regarding sexual function. In addition, the survey was carried out during COVID-19 pandemics. The use of an online platform enabled responding to the survey without the fears of exposure to the virus. These advantages may increase the response rate of our survey. When compared with the nationwide survey done in the USA (14) which had participation from 774 SCI individuals, the response to our current survey should be viewed as satisfactory for collecting replies from 74 SCI individuals and 13 caregivers.

### Upper limb function

For tetraplegic individuals, recovery of the arm/hand function, and upper trunk/body strength and balance were given the highest rankings. Deficits in the upper limb functions severely limit their independence in activities of daily living such as feeding, dressing, grooming and personal hygiene. In our survey, we found that tetraplegic individuals more than 3 years post-injury saw arm/hand function as a significantly higher priority than those who had injuries within 3 years. This might be related to their realization of the importance of upper limb function from their real life experience. Good upper trunk/body strength and balance is essential in providing a stable base for efficient movement of the arms and hands. This finding was in concordance with the systematic review by Simpson et al., where four of the five studies that included arm and hand function as an option among the functional recovery priorities of individuals with SCI, saw that to be the most desirable feature by tetraplegic individuals (35). Similar findings of high priority in recovery of upper limb function were also found in previous studies by Anderson (14), Lo et al. (36), and Agarwal et al. (17).

Efforts should be made to maintain optimal joint position and good ranges of motion with use of splintage (37), and active and passive exercises in order to maximize the remaining function of the upper limbs. Surgical reconstruction is another option for restoring upper limb function. However, several studies have shown that only a relatively small portion of tetraplegic individuals actually endeavor to receive upper limb reconstruction surgeries in real clinical practice (16, 38). It was estimated that only 14% of all appropriate surgical candidates had receive operations (39). Individuals with SCI are often reluctant to accept such procedures due to long post-operative rehabilitation periods and lack of social support. This is also in line with the local experience in Hong Kong. Timely patient education, strong medical and social support, and close interdisciplinary collaboration are essential to overcome these barriers to surgery (40).

### Bladder and bowel function

Neurogenic bladder and bowel dysfunction affect nearly all SCI individuals (41, 42), and they can lead to significant medical complications (43, 44) and reduced quality of life (45–47). In the previously mentioned systematic review by Simpson et al., restoration of bladder and bowel function was shown to be having the second highest priority in four out of five studies which showed results for tetraplegic, second to arm and hand functions (35). This order of prioritization, with arm and hand function ranked first, followed by bladder and bowel function, was also mirrored by a more recent study in India (17). The top prioritization for bladder and bowel function was seen amongst individuals with paraplegia in the systematic review by Simpson et al. (35), which concurs with our own findings. Furthermore, within the discussion on restoring bladder and bowel function, a recent survey identified bladder emptying as a top priority for restoring bladder function, and fecal continence as the top priority for restoring bowel function (48). Unfortunately, advances in the management for neurogenic bladder and bowel have been slow due to the difficulties in objective measurement of outcomes (49). The importance of the conditions are often overlooked because the impairments, unlike the obvious motor deficits, are less visible externally. Clinicians should be aware that they are highly ranked in the priority of functional recovery in both paraplegic and tetraplegic individuals.

### Walking

While “walking” might have been expected by most to be occupying a top priority, our study showed that in fact it was only ranked in the middle, together with elimination of chronic pain and normal sensation. The findings are not dissimilar to other studies (14, 15). An exception to this finding was described in one study which investigated the change in priorities at different stages of recovery. This particular study by Ditunno et al., showed that walking tends to be a higher priority of SCI individuals and clinicians only in the early stages of recovery (50). In our survey, we have also found that tetraplegic individuals place a significantly higher priority for walking movement only in the early years post-injury, and then gradually the priority for walking declines. This may suggest that individuals with SCI gradually cope and accept the use of wheelchair in daily activities. However, we also believe that smart city designs, availability of disabled access, disabled-friendly mass transit infrastructure, and community rehabilitation support will all have a bearing on SCI individuals' complacency to life-long wheelchair use. Furthermore, with the popularization of exoskeleton technology—both for rehabilitation and for personal use, this trend may change in the foreseeable future when advanced technology becomes more ubiquitously available. Yip et al. showed in a robust scoping review that there are numerous benefits to upright over-ground walking for chronic SCI individuals (28). These secondary biophysical benefits may also entice physicians and SCI individuals to adopt more walking exercises as a means to achieve life-long health maintenance. With the development of lightweight personal use exoskeletons with good safety profile, it is within expectation that this priority may gradually change with time (51).

### Sexual function

Physiological and psychosocial changes after SCI can significantly impact sexuality and sexual function (52, 53). Sexual function recovery was consistently rated as the least important among the 7 priority areas in all the 3 groups (tetraplegics, paraplegics, and caregivers) of participants in our study. These findings contradict with previous studies in Western countries. In UK and Netherlands, sexual function improvement was regarded as important as standing (15). And in the USA, it was ranked first by the paraplegic individuals and second by the tetraplegic individuals (14). Our results were more similar to a recent study conducted in India, in which sexual function was given lower ranking in the priority areas of recovery (17). We believe this may be related to the difference between Asian and Western culture when discussing the various aspects of sexual intimacy. In particular for the Chinese, where Confucianism has been the primary rationalistic religion and philosophy for over 2,000 years, open discussion about sexuality is often considered as taboo and is discouraged or even forbidden. Indeed, studies have shown that even in a normal Chinese population, the different aspects of Confucian culture has a notable effect on sexual behavior (54). Another study comparing the life satisfaction in persons with SCI between Japan and Sweden had also found Japanese SCI individuals to be less sexually satisfied and partially attributed the finding to the predominant Confucian cultural influences present in East Asia (55). Further investigation in this area can give us a better understanding of this observation. From our own experience of SCI rehabilitation in Hong Kong, sexual rehabilitation is still very often neglected by the SCI community and clinicians.

### Community rehabilitation service needs, and advance in technology and research

This study also highlighted the increasing demand for community rehabilitation service for SCI. Both individuals with SCI and caregivers thought that such services should be of greater availability. Individuals with SCI and caregivers also expected advancements in technology and research to bring them further improvements in quality of life in the future. This appears to be in contrast with another prospective study on SCI which was carried out in Hong Kong about 10 years ago. Chan and Chan noted that high-end technology is not as well-received in Chinese populations as they are in Western countries (56). The same study also discussed the need to balance the desire for maximization of independence, with respect for the personal preference of individuals, which in the case of Chinese people, tend to prefer human assistance to technical support (56). The difference may be a reflection of significant advance, wider availability, and better affordability of technology in the recent decade, like smartphones and smart home appliances which can easily be configured to suit the needs of individuals with SCI. The ubiquitous presence of information on the internet about research which may enhance the recovery and quality of life has also brought hope to many individuals with SCI and their caregivers, and made them more optimistic.

The results of our survey show that SCI individuals and caregivers do have high expectations for the advancements in robotics and technology in improvement of their quality of life. However, clinicians and researchers of SCI rehabilitation appeared to be less concerned about evaluation of the use of assistive device and robotics (34). Though advanced technology has become more available, the actual number of SCI individuals who have access to exoskeleton rehabilitation is still relatively small compared to the whole SCI community, and as a result we believe that meaningful user feedback to the engineers of said technology is still tremendously lacking. A high cost of research and development leading to poor user uptake may lead to a vicious cycle of low level of inclusion. We strongly believe that the actual needs of the SCI individuals and how they can utilize these advances in the community needs further study. The limitations in technology are also somewhat less discussed in literature. We suggest that clinicians and researchers should include these parameters as important rehabilitation outcomes. Government funding in Hong Kong usually only support purchase of the basic model of essential assistive devices. In addition to the resource being put into development advanced technologies, it is time that the healthcare and welfare authorities reconsider the funding or reimbursement policy so that the results of such advance can be enjoyed by individuals with SCI and improve their quality of life.

### Limitations

One limitation of requesting individuals with SCI to rank their functional recovery priorities in an integral manner, is the inability to assign relative weights for different impairments. For the same respondent, it was not feasible for us to understand the relative importance of various functions to him- or herself. More sophisticated methods like choice-based evaluation with time trade-off technique (57) or discrete-choice experiment (36, 58) have been used to overcome part of these limitations. For example, to determine the preference for upper limb reconstruction surgery in tetraplegic subjects compared with treatment preference of 3 other impairment areas, Snoek et al. used a utility score is calculated based on the time trade-off techniques (57). The utility scores of tetraplegia without impairment of upper limb function, bladder and bowel function, standing and walking, and sexual function were compared to determine the difference in preference. This method may give us a better understanding of the actual situation. However, from our observation, this method appears to be too complicated for the self-administered online platform used in this study.

Secondly, sampling bias may lead to a skew in the responses specific to the demographic of this particular cohort of respondents. This may present a problem in the analysis of subgroups according to gender, time post-injury and age. Since some of the subgroups (paraplegic individuals who were female, 3 years or less post-injury, and age 40 or younger) had case number <10, we are not able to tell whether the lack of statistically significant difference in some priority areas are genuine or due to the small sample sizes. Future studies with larger sample sizes would be needed to overcome this problem.

## Conclusion

This survey is the first study aimed at identifying the priorities of functional recovery amongst SCI individuals of Chinese ethnicity and cultural background. This is also the first such study to survey the opinions and priorities of primary caregivers of SCI individuals. Data on the utilization and opinions of community rehabilitation services, and views on advances in technology and research are also presented. The information provides a better understanding of the views and needs of the SCI community. It will be a useful reference for planning of future research and provision of rehabilitation services in Hong Kong and other parts of China.

## Data availability statement

The data presented in the study are deposited in the HKU Data Repository https://datahub.hku.hk/. The dataset can be accessed at https://doi.org/10.25442/hku.19588408.

## Ethics statement

The studies involving human participants were reviewed and approved by Institutional Review Board of the University of Hong Kong/Hospital Authority Hong Kong West Cluster. The participants provided their written informed consent to participate in this study.

## Author contributions

CL, PK, and IS: concept or design and acquisition of data. CL, PK, and CY: analysis or interpretation of data and drafting of the article. YH, YW, and KC: critical revision for important intellectual content. All authors contributed to the article and approved the submitted version.

## Funding

This research was generously supported by the Get Up and Walk Campaign of the Department of Orthopaedics and Traumatology, School of Clinical Medicine, Li Ka Shing Faculty of Medicine, The University of Hong Kong.

## Conflict of interest

The authors declare that the research was conducted in the absence of any commercial or financial relationships that could be construed as a potential conflict of interest.

## Publisher's note

All claims expressed in this article are solely those of the authors and do not necessarily represent those of their affiliated organizations, or those of the publisher, the editors and the reviewers. Any product that may be evaluated in this article, or claim that may be made by its manufacturer, is not guaranteed or endorsed by the publisher.
