# Supplementary material for: Functional recovery priorities and community rehabilitation service preferences of spinal cord injury individuals and caregivers of Chinese ethnicity and cultural background
Source: Front Neurol. 2022 Aug 3;13:941256. doi: 10.3389/fneur.2022.941256 (PMC9382587; doi:10.3389/fneur.2022.941256)
Supplement: Supplementary file 1 [file Data_Sheet_1.docx]

**Appendix 1**

A survey on the priorities of functional recovery and available rehabilitation service in spinal cord injury community

脊髓損傷病人及照顧者對功能復原期望的優先次序及現有的社區復康服務意見調查

**Investigator:**

Dr. Lam Chor Yin, Department of Orthopaedics and Traumatology, The University of Hong Kong

研究負責人

林楚賢醫生 - 香港大學矯形及創傷外科系

You are invited to participate in the survey on the priorities of functional recovery and rehabilitation service in spinal cord injury community, a research study to understand the needs of the SCI community better. Your participation is voluntary. It will not affect your rights or opportunities in using medical or other services in the future.

你被邀請參加脊髓損傷病人及照顧者對功能復原期望的優先次序及現有的社區復康服務意見調查。

本調查希望能夠使臨床醫護及研究人員對脊髓損傷病人及照顧者的意見有更佳的了解。本調查的參與是自願性的，它不會影響你將來使用醫療或其他服務的權利和機會。

**Confidentiality**

Results of this study may be presented at meetings or in publications. However, information identifying you will not be given without your permission. We will not use identifying information or personal data (such as your name) on any published reports or articles. All collected information will be kept strictly confidential. According to Hong Kong Law Chapter 486 the Personal Data (Privacy) Ordinance, you have the right to keep your data confidential. If you have any concern on personal data collection in this study or related to this study regarding collection, saving, management, control, use (analysis or comparison), transfer outside Hong Kong, confidential, delete and / or any manner. For further information or enquiry, please contact Office of the Privacy Commissioner at 2827-2827.

保密

本研究的結果可在會議或出版物中提出。但是，未經您的許可，不會提供識別您身份的資料。我們不會在任何已發布的報告或文章上使用個人資料（如您的姓名）。所有在研究期間收集的資料將被嚴加保密。根據「個人資料（私隱）條例」，香港法律第486章， 您有權對您個人資料進行保密。如對本項研究中或與本項研究有關的個人資料的收集、保管、保留、管理、控制、使用（分析或比較）、在香港內外轉讓、保密、刪除和/或任何方式處理有任何問題，您可以諮詢個人資料私隱私隱專員公署（電話號碼：2827 2827）。

This study was reviewed by Institutional Review Board of the University of Hong Kong / Hospital Authority Hong Kong West Cluster and it is one of the authorized parties to access the subjects’ records related to the study for ethics review purpose. For further information or enquiry regarding patient’s right as a participant in this study, please contact Institutional Review Board of the University of Hong Kong/Hospital Authority Hong Kong West Cluster at 2255-4086.

For further information or enquiry

Please contact research coordinator Dr. Lam Chor Yin via 2255-5228

這項研究已經通過香港大學及醫管局港島西醫院聯網研究倫理委員會的審查。香港大學及醫管局港島西醫院聯網研究倫理委員有權審查本項研究有關之記錄作研究審查的目的。如對參加本研究的病人權益有任何查詢, 請聯絡香港大學及醫管局港島西醫院聯網研究倫理委會 (電話: 2255 4086) 。

查詢

如果您對本研究有任何查詢, 請聯絡林楚賢醫生(電話: 2255 5228) 。

- 本人年滿18歲，能看懂書面中文或英文。

I am 18 or above, and I can understand written Chinese or English.

- 本人同意參與脊髓損傷病人及照顧者對功能復原期望的優先次序及現有的社區復康服務意見調查

I consent to participate in the survey on the priorities of functional recovery and available rehabilitation service in spinal cord injury community.

- 本人同意調查負責人使用是次調查獲取的資料進行現時及將來的研究

I consent the investigator to use the data obtained in this survey for current and future research.

Background information and functional recovery priorities

基本資料及功能復原的優先次序

1. Are you an individual with spinal cord injury or the primary caregiver? 你是脊髓損傷患者還是主要照顧者?

Individual with injury 患者/ primary caregiver主要照顧者 🡪Q.3

Gender 性別:

Age 年齡 :

1. What year did the spinal cord injury occur? 脊髓損傷發生的年份



Occurred in ____ 在 _____年發生

Patient 🡪 Q.4

1. How many years have you been taking care of the patient? 你照顧了患者多少年？

_____ Years/ _______年

1. What level is the spinal cord injury? 脊髓損傷的位置 ?

Cervical 頸椎/ Thoracic胸椎/ Lumbosacral腰,骶椎/ Don’t know 不知道

1. Which parts of the body are paralyzed? 癱瘓的身體部分?

Tetraplegic四肢癱瘓/ Paraplegic 下肢癱瘓

1. What is the severity of the spinal cord injury? 脊髓損傷的嚴重程度？

Complete完全 / Incomplete 不完全 / Don’t know不知道

1. What is the current primary method of mobility? 日常主要的活動能力及模式?

Bedbound 臥床

Powered wheelchair 電動輪椅

Manual wheelchair 手動輪椅

Wheelchair controlled or propelled by the caregiver 由照顧者控制或推動的輪椅

Walk with aids and assistance 在他人協助下使用助行器具步行

Walk with aids independently不需要協助,使用助行器具步行

Walk without assistance or aids 不需要協助或助行器具,可以獨立步行

1. What is the current residence? 現時居所

私人房屋 Private housing

公共房屋 Public housing

私人安老院/私人院舍 Private home for the aged/ institution

政府或資助安老院/院舍 Government or subvented home for the aged/ institution

過渡性住宿 （例如：新頁居）Transitional housing (e.g New Page Inn)

其他 Others:

1. How many hours per day on average the primary caregiver spends on taking care of the patient? If no assistance from caregiver is required, please input “0”. 平均每天主要照顧者提供照顧的時數？如果不需要他人照顧，請填「0」。

_______ hours/ _______小時

1. 每週平均做運動的時數(自己鍛煉或者由你的照顧者協助，不包括社區復康服務)？

How many hours per week on average do you exercise (including exercise by yourself, or assisted by your caregiver; excluding community rehabilitation service)?

_______ hours/ _______小時

1. 每週花多少小時離開住所到外面進行休閒活動（不包括覆診或社區復康服務）?

How many hours per week on average do you leave your residence for leisure activities (excluding medical follow-ups and community rehabilitation service)?

_______ hours/ _______小時

1. What gain of function would be important for you? 你認為那類功能改善是重要的

Rank the following functional recovery in order of importance to you, 1 being most important and 7 being least important:


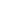


請按重要性為下列功能恢復排列優先次序,1 為最重要, 7為最不重要:

A) arm/hand function 手臂及手部功能

B) upper body/trunk strength and balance上身及軀幹的力度及平衡

C) bladder/bowel function 膀胱及大便功能

D) sexual function 性功能

E) elimination of chronic pain消除慢性疼痛

F) normal sensation正常的感覺功能

G) walking movement步行

Use of community rehabilitation service

社區復康服務的使用

1. Do you think community rehabilitation service is important? 你覺得社區復康服務重要嗎?

Very important非常重要/ Fairly important頗重要/ Important重要 / Slightly important稍微重要/ Not important不重要/ No opinion無意見

1. Do you think the current level of community rehabilitation service is adequate?你認為現有的社區復康服務足夠嗎？

Yes 足夠/ No opinion無意見🡪 Q. 16/ No 不足夠

15. What kind of community rehabilitation service should be strengthened? 那一種復康服務需要加強？(可選多過一項)

Physiotherapy物理治療

Occupational therapy職業治療

Speech therapy言語治療

Nursing護理服務

General care service一般照顧服務

Others其他: ________

16. Currently using any community rehabilitation service? 現時有使用社區復康服務嗎?。

Yes/ No有/沒有

No 🡪 Q.19

17. Which organization provides such community rehabilitation service? 使用的社區復康服務是由甚麼機構提供?

Hospital Authority – Community rehabilitation service醫院管理局 - 社區復康服務(上門)

Hospital Authority – Outpatient/ Day rehabilitation service醫院管理局 - 門診/日間復康服務

Social Welfare Department/NGO – Home Care Service社會福利署/非政府組織- 家居護理服務

Social Welfare Department/NGO – Outpatient/ Day Care Service社會福利署/非政府組織- 門診/日間復康服務

Private私人服務

Others其他:___________________________

18. What kinds of community rehabilitation service are you currently using? (can choose more than one) 現在正接要那一種復康服務(可選多過一項)

Physiotherapy物理治療

Occupational therapy職業治療

Speech therapy言語治療

Nursing護理服務

General care service一般照顧服務

Others其他: ________

🡪 Q.21

19. Would you use community rehabilitation service if it is available？如果有社區復康服務提供給你，你會使用嗎？

Yes會/可能會 Maybe/ No不會 🡪 Q.20

20. What community rehabilitation service would you like to use? (can choose more than one)你會使用那一些社區復康服務 (可選多過一項)

Physiotherapy物理治療

Occupational therapy職業治療

Speech therapy言語治療

Nursing護理服務

General care service一般照顧服務

Others: _______其他

Expectations for advanced technology and research

對科技和研究的期望

21. If advanced technology can be provided to you (e.g. robotic rehabilitation, smart home modifications), would you like to use it? 如果可以提供一些高科技服務及器材（如機械腳復康，智能家居改裝），你會使用嗎？

Yes會/ No不會/ Don’t know不知道

22. How likely do you think the advance in technology and research will significantly change the quality of life in spinal cord injury community in the coming 10 years? 你覺得科技及研究的進步在未來十年可以對脊髓損傷患者或其照顧者的生活質素有重大改善的機會有多大？

Unlikely (<20% chance)/ somewhat likely (20-40% chance) / likely (40-60% chance)/ fairly likely (60-80% chance) / very likely (>80% chance)

不大可能（少過百分之二十的機會）/有些可能(百分之二十至四十的機會)/有可能(百分之四十至六十的機會)/頗大可能(百分之六十至八十的機會)/非常可能(大過百分之八十的機會)

Name (Optional)姓名(選填):

Contact number (Optional) 聯絡電話(選填):

Would you like to be contacted for further interview or research? Yes/ No

如有需要，你希望我們就進一步的訪談及研究聯絡你嗎？想/不想

*The End*
